# Validation of Type 2 Diabetes Risk Variants Identified by Genome-Wide Association Studies in Han Chinese Population: A Replication Study and Meta-Analysis

**DOI:** 10.1371/journal.pone.0095045

**Published:** 2014-04-15

**Authors:** Yi-Cheng Chang, Pi-Hua Liu, Yu-Hsiang Yu, Shan-Shan Kuo, Tien-Jyun Chang, Yi-Der Jiang, Jiun-Yi Nong, Juey-Jen Hwang, Lee-Ming Chuang

**Affiliations:** 1 Department of Internal Medicine, National Taiwan University Hospital HsinChu branch, Taipei, Taiwan; 2 Institute of Biomedical Science, Academia Sinica, Taipei, Taiwan; 3 Clinical Informatics and Medical Statistics Research Center, College of Medicine, Chang Gung University, Taoyuan, Taiwan; 4 Department of Internal Medicine, National Taiwan University Hospital, Taipei, Taiwan; 5 Institute of Clinical Preventive Medicine, College of Public Health, National Taiwan University, Taipei, Taiwan; Tor Vergata University of Rome, Italy

## Abstract

**Background:**

Several genome-wide association studies (GWAS) involving European populations have successfully identified risk genetic variants associated with type 2 diabetes mellitus (T2DM). However, the effects conferred by these variants in Han Chinese population have not yet been fully elucidated.

**Methods:**

We analyzed the effects of 24 risk genetic variants with reported associations from European GWAS in 3,040 Han Chinese subjects in Taiwan (including 1,520 T2DM cases and 1,520 controls). The discriminative power of the prediction models with and without genotype scores was compared. We further meta-analyzed the association of these variants with T2DM by pooling all candidate-gene association studies conducted in Han Chinese.

**Results:**

Five risk variants in *IGF2BP2* (rs4402960, rs1470579), *CDKAL1* (rs10946398), *SLC30A8* (rs13266634), and *HHEX* (rs1111875) genes were nominally associated with T2DM in our samples. The odds ratio was 2.22 (95% confidence interval, 1.81-2.73, *P*<0.0001) for subjects with the highest genetic score quartile (score>34) as compared with subjects with the lowest quartile (score<29). The incoporation of genotype score into the predictive model increased the C-statistics from 0.627 to 0.657 (*P*<0.0001). These estimates are very close to those observed in European populations. Gene-environment interaction analysis showed a significant interaction between rs13266634 in *SLC30A8* gene and age on T2DM risk (*P*<0.0001). Further meta-analysis pooling 20 studies in Han Chinese confirmed the association of 10 genetic variants in *IGF2BP2*, *CDKAL1*, *JAZF1*, *SCL30A8*, *HHEX*, *TCF7L2*, *EXT2*, and *FTO* genes with T2DM. The effect sizes conferred by these risk variants in Han Chinese were similar to those observed in Europeans but the allele frequencies differ substantially between two populations.

**Conclusion:**

We confirmed the association of 10 variants identified by European GWAS with T2DM in Han Chinese population. The incorporation of genotype scores into the prediction model led to a small but significant improvement in T2DM prediction.

## Introduction

Type 2 diabetes mellitus (T2DM) is a complex disease influenced by both genetic and environmental factors. The heritability of T2DM is relatively strong with an estimated *h* ^2^ of 31–69% [1]. Previous genetic studies have suggested the involvement of multiple genes with modest effects in the pathogenesis of T2DM [2]. This notion was supported by several genome-wide association studies (GWAS) for T2DM in European population [3]–[7]. These GWAS showed associations of approximately ∼40 risk variants with T2DM in European population. Further large-scaled meta-analyses confirmed these associations and estimated their relative contributions in European populations [8]. These discoveries greatly advanced our understanding toward the genetic architecture of T2DM and provided valuable tools for the prediction of personal T2DM risk in European populations.

The prevalence of diabetes mellitus has increased rapidly in Chinese populations in recent decades. In 2013, the prevalence of diabetes and prediabetes was estimated to be 11.6% and 50.1% respectively, suggesting that there were 113.9 million Chinese adults with diabetes and 493.4 million with prediabetes [9]. This dramatic surge of T2DM prevalence poses a serious threat to the public health of Chinese populations. Since diabetes can be effectively prevented by life-style or pharmacological intervention in high-risk patients, it is important to identify high-risk subjects for preventive measures. With the strong heritability of diabetes, genetic information is expected to offer additional benefits towards the identification of high-risk subjects. Previous studies incorporating genetic scores into T2DM prediction models have successfully demonstrated the benefit of utilizing such approach [10], [11]. However, this approach has not yet been validated in Han Chinese population. Given the heterogeneous genetic structures between European and Chinese populations, it is essential to confirm the association and the predictive value of these genetic variants in the Chinese.

In this case-control study, we genotyped 24 risk variants identified from European GWAS in 3,040 Han Chinese. The associations of these variants with T2DM were analyzed in our sample and were further validated by a meta-analysis pooling 20 case-control association studies of Han Chinese. The discriminative power of the prediction models with and without genotype information were then compared.

## Materials and Methods

### Study participants

A total of 760 T2DM patients were recruited from the metabolism clinics of the National Taiwan University Hospital (NTUH) and another 760 T2DM patients were recruited from the metabolism clinic of the Yunlin branch of NTUH. T2DM were diagnosed according to the criteria of the American Diabetes Association [12] or the use of anti-diabetic therapy. Patients with ages of onset below 35 years were excluded. In addition, 760 glucose-tolerant healthy controls were recruited from the health check-up service of NTUH and another 760 controls were recruited from a community screening for metabolic syndrome in the Yunlin county of Taiwan. Glucose tolerance was defined as fasting plasma glucose < 126 mg/dl or 2-hour plasma glucose < 200 mg/dl during a 75-g oral glucose tolerance test (OGTT). Written informed consent was obtained from each participating subject, and the study was approved by the institutional review board of the National Taiwan University Hospital.

### Selection of SNPs and genotyping

Twenty-four genetic risk variants were selected from GWAS or well-established candidate-gene association studies for T2DM in European populations [3]–[7]. In view of the low risk allele frequencies and the negative T2DM association of rs7903416 in the *TCF7L2* gene observed in previous researches in Chinese population [13], [14], we genotyped another SNP in this gene, rs290487, which has been reported to be associated with T2DM in Chinese [13]. Genotype data of rs7903146 were retrieved from our previous study [13]. Genotyping was performed using the GenomeLab SNPstream genotyping platform (Beckman Coulter) and its accompanying SNPstream software suite. The concordance rate based on this platform was 99.62% [15].

### Search criteria for meta-analysis

We searched the PubMed database with the keywords “genetic”, “association”, “genome”, “genome-wide association study”, “type 2 diabetes mellitus”, “type 2 diabetes”, “diabetes”, “Chinese”, “Taiwanese”, and “Han Chinese” before October 2013. Eligible criteria included (1) candidate-gene association studies for T2DM published in peer-reviewed journal, (2) participants recruited from independent populations, (3) genotype information sufficient to calculate allele counts, (4) candidate genes tested in our study and (4) study population restricted to Han Chinese. A total of 21 studies that fulfill were indentified [13], [16]–[35]. Full-text manuscripts were obtained for all studies. One study was excluded because its study population was confined to She Chinese population[35]. Data extraction was performed and examined independently by two reviewers (YCC and YHY).All procedures were conformed to the Preferred Reporting Items for Systemic Review and Meta-Analysis (PRISMA) guidelines for meta-analysis[36]. The PRISMA check list and flow diagram was shown in Checklist S1.

### Statistical analyses

Hardy-Weinberg equilibrium (HWE) test was performed for each SNP in the control group before marker-trait association analysis. Tests for the associations of each SNP and haplotype with type 2 diabetes were conducted using logistic regression. Nominal two-sided *P*-values were reported. Multivariate analysis with age, gender, and BMI as covariates was performed using multivariate logistic regression. The odds ratio (OR) and 95% confidence interval (CI) associated with each risk allele were also estimated. Pairwise gene-gene and gene-environment interactions were analyzed by logistic regression. The significance of interaction was adjusted for multiple testing using the Bonferroni method.

To test the cumulative effects of genetic variants on the T2DM risk, weighted genetic score for each risk allele was calculated using the beta-coefficients of logistic regression model. All participants were divided into four equal groups according to their genetic score (<29, 29–31, 31–34, >34). The OR and 95% CI for each group were estimated using the lower quartile group (score<29) as the reference group. The statistical power of this study for each SNP was estimated using the Genetic Power Calculators (http://pngu.mgh.harvard.edu/~purcell/gpc/) assuming diabetes prevalence of 8% [37]. Meta-analysis under fixed effect models were used to estimate pooled odds ratio (OR) using the Comprehensive Meta-Analysis software (Biostat, Englewood, NJ). Cochran's Q test and *I* ^2^ was used to assess heterogeneity between the individual studies. The Z test was used to determine the significance of the pooled OR.

## Results

### Characteristics of study subjects and SNP information

Twenty four SNPs were successfully genotyped in 1,502 unrelated T2DM cases and 1,518 glucose-tolerant controls except for rs10811661 in the *CKD2A/B* gene, in which genotyping failed in all samples. The demographic and biochemical characteristics of the study participants are shown in Table 1. Basic information of these SNPs is summarized in Table 2. All SNPs were in Hardy-Weinberg equilibrium (Table 2). The average call rate was 99.08%.

**Table 1:** Characteristics of study participants.

	Case	Control	*P* value
Number	1502	1518	
Age (year)	60.42±11.83	55.83±15.83	<0.0001
Male sex (%)	51.26	50.86	0.82
Body mass index (kg/m^2^)	25.45±4.27	24.27±3.66	<0.0001
Fasting glucose (mg/dL)	157.00±54.87	93.84±17.27	<0.0001
Triglycerides (mg/dL)	171.82±88.85	115.76±77.99	<0.0001
Total cholesterol (mg/dL)	195.02±50.13	197.18±38.15	0.20

**Table 2:** Information of SNP.

	SNP rs#	Gene	Chr.	Chr. position	Gene region	Allele		Risk allele	Controls HW *P*	Call Rate (%)
						Minor	Major			
1	rs2641348	*ADAM30*	1	120149926	Exon L [Leu] ⇒ P[Pro]	C	T	C	1	99.5
2	rs10923931	*NOTCH2*	1	120319472	intron	T	G	T	1	99.7
3	rs7578597	*THADA*	2	43644474	Exon T [Thr] ⇒ A[Ala]	C	T	T	1	99.9
4	rs10490072	*BCL11A*	2	60581582	intergenic	C	T	T	1	99.1
5	rs4402960	*IGF2BP2*	3	186994389	intron	T	G	T	0.45	98.4
6	rs1470579	*IGF2BP2*	3	187011782	intron	C	A	C	0.65	98.6
7	rs17036101	*SYN2,PPARG*	3	12252845	intergenic	A	G	G	0.64	99.6
8	rs4607103	*ADAMTS9*	3	64686934	upstream	T	C	C	0.91	99.1
9	rs10010131	*WFS1*	4	6410977	intron	A	G	G	1	99.4
10	rs6446482	*WFS1*	4	6413755	intron	C	G	G	0.54	99.4
11	rs10946398	*CDKAL1*	6	20769003	intron	C	A	C	0.42	99
12	rs9472138	*VEGFA*	6	43919730	intergenic	T	C	C	0.74	99
13	rs864745	*JAZF1*	7	27953796	intron	G	A	A	0.23	98.6
14	rs13266634	*SLC30A8*	8	118253964	Exon R [Arg] ⇒W[Trp]	T	C	C	0.35	99.2
15	rs1111875	*HHEX*	10	94452862	downstream	C	T	C	0.80	99.3
16	rs7923837	*HHEX*	10	94471897	intergenic	G	A	G	0.79	98.7
17	rs7903146	*TCF7L2*	10	114748340	intron	T	C	T		
18	rs290487	*TCF7L2*	10	114899720	intron	C	T	C	0.91	99.8
19	rs7480010	*LOC387761*	11	42203294	intergenic	G	A	G	0.938	97.4
20	rs1113132	*EXT2*	11	44209979	intron	G	C	C	0.82	98.7
21	rs11037909	*EXT2*	11	44212190	intron	C	T	T	0.95	99.5
22	rs3740878	*EXT2*	11	44214378	intron	G	A	A	1	99.2
23	rs1153188	*DCD*	12	53385253	intergenic	T	A	A	1	99.5
24	rs7961581	*TSPAN8,LGR5*	12	69949359	intron	C	T	C	0.93	98.2
25	rs8050136	*FTO*	16	52373766	Intron	A	C	A	0.24	99.7

Chr., chromosome; HW-*P*, P-value for Hardy-Weinberg equilibrium.

### Single-locus SNP association analysis

**Figure 1:**
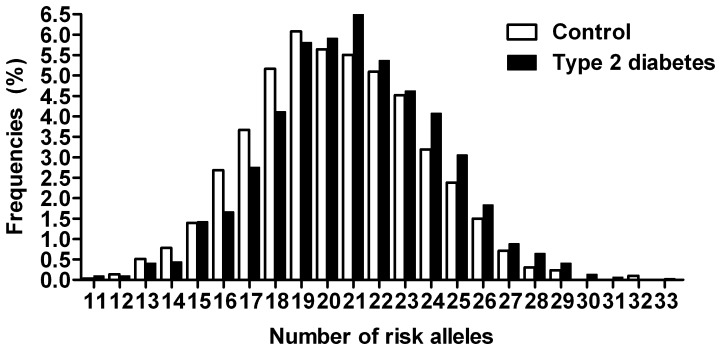
Distribution of risk allele frequencies in type 2 diabetic cases and controls.

The results of SNP association analysis with T2DM are listed in Table 3. Among all SNPs, six SNPs in five genes were nominally associated with T2DM, including rs4402960 in *IGFBP2* (OR, 1.13; 95% CI, 1.033–1.257, *P* = 0.013), rs1470579 in *IGFBP2* (OR, 1.1; 95%CI, 1.006–1.220, *P* = 0.049), rs10946398 in *CDKAL1* (OR, 1.14; 1; 95% CI, 050–1.249; *P* = 0.005), rs13266634 in *SLC30A8* (OR, 1.22; 95% CI, 1.127–1.334; *P*<0.0001), rs1111875 in *HHEX* (OR, 1.19; 95% CI, 1.088–1.303; *P*<0.0001), and rs1153188 in *DCD* (OR, 1.55; 95% CI, 1.107–2.18, *P* = 0.02) genes. The directions of association are consistent with those reported in European GWAS [3]–[8] except for rs1153188 in the *DCD* gene. The reported risk A allele at rs1153188 in European population was associated with reduced T2DM risk in our study. The numbers of risk alleles carried by T2DM cases were significantly more than those carried by the controls (20.94 vs. 20.35 alleles, *P*<0.0001) (Figure 1).

**Table 3:** SNP association with type 2 diabetes.

#	SNP	Gene	MAF Cases Control		OR (adjusted OR)*	95% CI (adjusted 95% CI)*	*P (*adjusted *P*)*	Power (%) (adjusted Power) *
1	rs2641348	*ADAM30*	0.0306	0.0297	1.018 (1.029)	0.757–1.368 (0.762–1.389)	0.91 (0.85)	5.19 (5.49)
2	rs10923931	*NOTCH2*	0.0302	0.0288	1.040 (1.053)	0.772–1.402 (0.778–1.426)	0.79 (0.73)	5.91 (6.59)
3	rs7578597	*THADA*	0.0033	0.0066	2.00 (1.618)	0.933–4.29 (0.745–3.514)	0.075 (0.22)	80.9 (46.4)
4	rs10490072	*BCL11A*	0.001	0.0013	1.346 (1.695)	0.300–6.024 (0.375–7.656)	0.69 (0.49)	7.80 (15.2)
5	rs4402960	*IGF2BP2*	0.2551	0.2267	1.168 (1.166)	1.036–1.316 (1.033–1.317)	**0.011 (0.013)**	80.4 (79.5)
6	rs1470579	*IGF2BP2*	0.2611	0.2385	1.128 (1.127)	1.002–1.269 (1.000–1.271)	**0.046 (0.049)**	60.1 (59.3)
7	rs17036101	*SYN2,PPARG*	0.0309	0.0304	0.983 (0.976)	0.757–1.366 (0.724–1.318)	0.91 (0.87)	5.17 (5.02)
8	rs4607103	*ADAMTS9*	0.3401	0.3334	0.970 (0.975)	0.870–1.081 (0.873–1.089)	0.59 (0.65)	9.29 (7.87)
9	rs10010131	*WFS1*	0.0599	0.0613	1.022 (1.014)	0.831–1.26 (0.822–1.252)	0.83 (0.89)	5.57 (5.23)
10	rs6446482	*WFS1*	0.0664	0.0694	1.047 (1.038)	0.859–1.277 (0.849–1.269)	0.65 (0.71)	7.91 (6.40)
11	rs10946398	*CDKAL1*	0.3908	0.3527	1.180 (1.167)	1.061–1.312 (1.048–1.299)	**0.002 (0.005)**	92.2 (88.3)
12	rs9472138	*VEGFA*	0.1377	0.1337	0.968 (0.968)	0.834–1.123 (0.832–1.126)	0.66 (0.67)	7.59 (7.59)
13	rs864745	*JAZF1*	0.2111	0.2237	1.074 (1.084)	0.950–1.212 (0.958–1.227)	0.25 (0.2)	24.3 (30.2)
14	rs13266634	*SLC30A8*	0.4257	0.4924	1.310 (1.302)	1.182–1.451 (1.173–1.446)	**<0.0001 (<0.0001)**	99.9 (96.8)
15	rs1111875	*HHEX*	0.329	0.2895	1.199 (1.213)	1.075–1.337 (1.085–1.355)	**<0.0001 (<0.0001)**	94.1 (96.6)
16	rs7923837	*HHEX*	0.2054	0.188	1.113 (1.126)	0.980–1.264 (0.990–1.282)	0.098 (0.071)	43.96 (50.8)
17	rs7903146	*TCF7L2*	0.0234	0.0287	0.81 (0.89)	0.500–1.310 (0.55–1.46)	0.36 (0.66)	98.3 (60.5)
18	rs290487	*TCF7L2*	0.3965	0.3810	1.069 (1.076)	0.962–1.187 (0.967–1.197)	0.22 (0.18)	27.4 (32.6)
19	rs7480010	*LOC387761*	0.2196	0.2163	1.018 (1.024)	0.901–1.15 (0.904–1.159)	0.77 (0.71)	6.12 (6.98)
20	rs1113132	*EXT2*	0.411	0.4178	0.972 (0.979)	0.876–1.078 (0.881–1.089)	0.59 (0.69)	8.93 (7.24)
21	rs11037909	*EXT2*	0.4178	0.4252	1.031 (1.022)	0.874–1.075 (0.920–1.135)	0.56 (0.68)	9.67 (7.36)
22	rs3740878	*EXT2*	0.4183	0.4200	1.007 (1.001)	0.895–1.101 (0.900–1.112)	0.89 (0.99)	5.24 (5.05)
23	rs1153188	*DCD*	0.0193	0.0113	0.583 (0.602)	0.380–0.892 (0.392–0.925)	**0.013 (0.02)**	79.1 (73.3)
24	rs7961581	*TSPAN8,LGR5*	0.2311	0.2332	0.987 (0.966)	0.874–1.114 (0.854–1.093)	0.83 (0.58)	5.62 (9.48)
25	rs8050136	*FTO*	0.1268	0.1244	1.024 (1.018)	0.877–1.195 (0.869–1.192)	0.76 (0.82)	6.27 (5.72)

MAF, minor allele frequency; OR: odds ratio; 95% CI, 95% confidence interval; * Associated with risk alleles adjusted for age, sex, and BMI.

### Genotype score and diabetes risk

**Figure 2:**
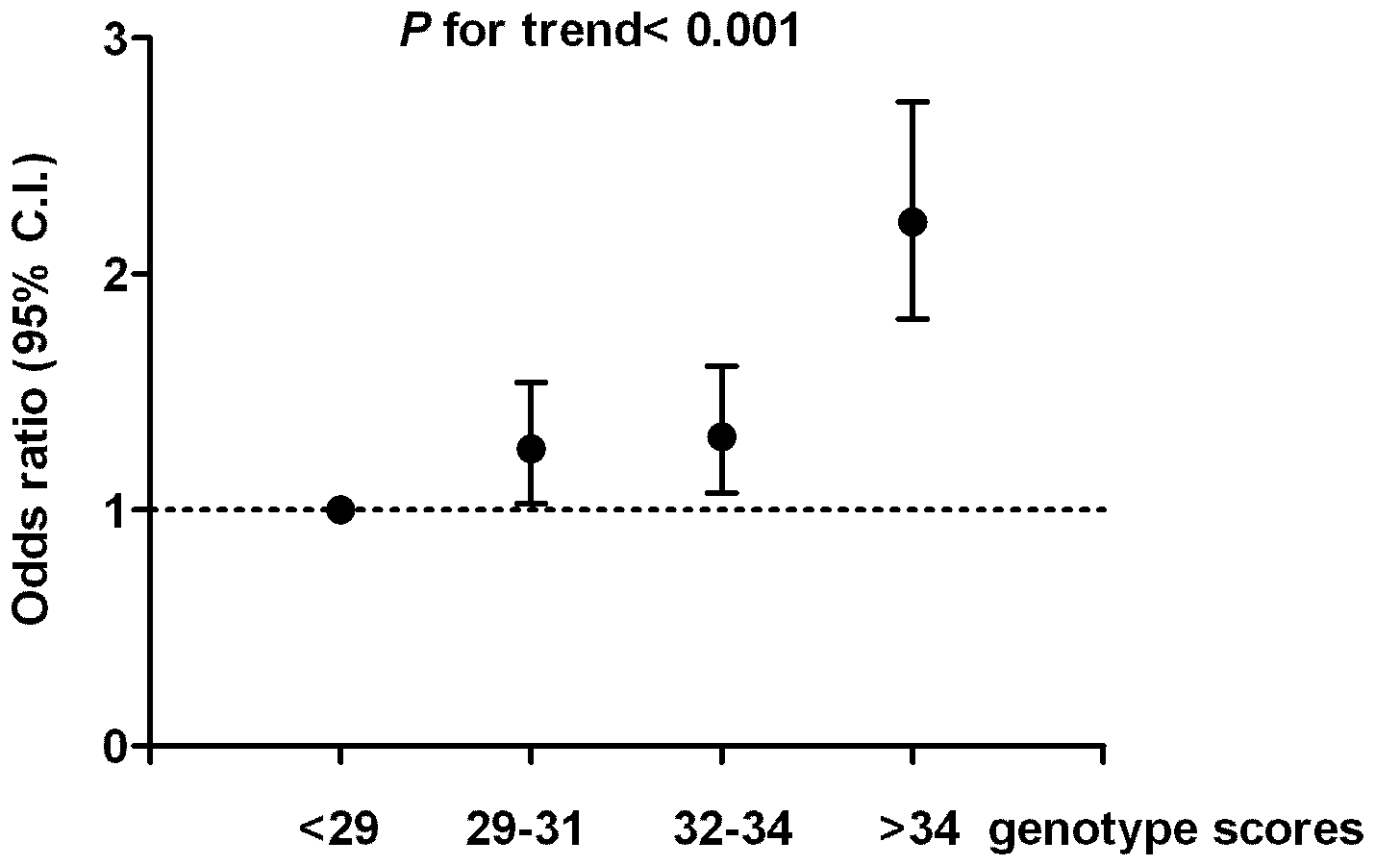
Odds ratio of type 2 diabetes according to weighted genetic scores.

**Figure 3:**
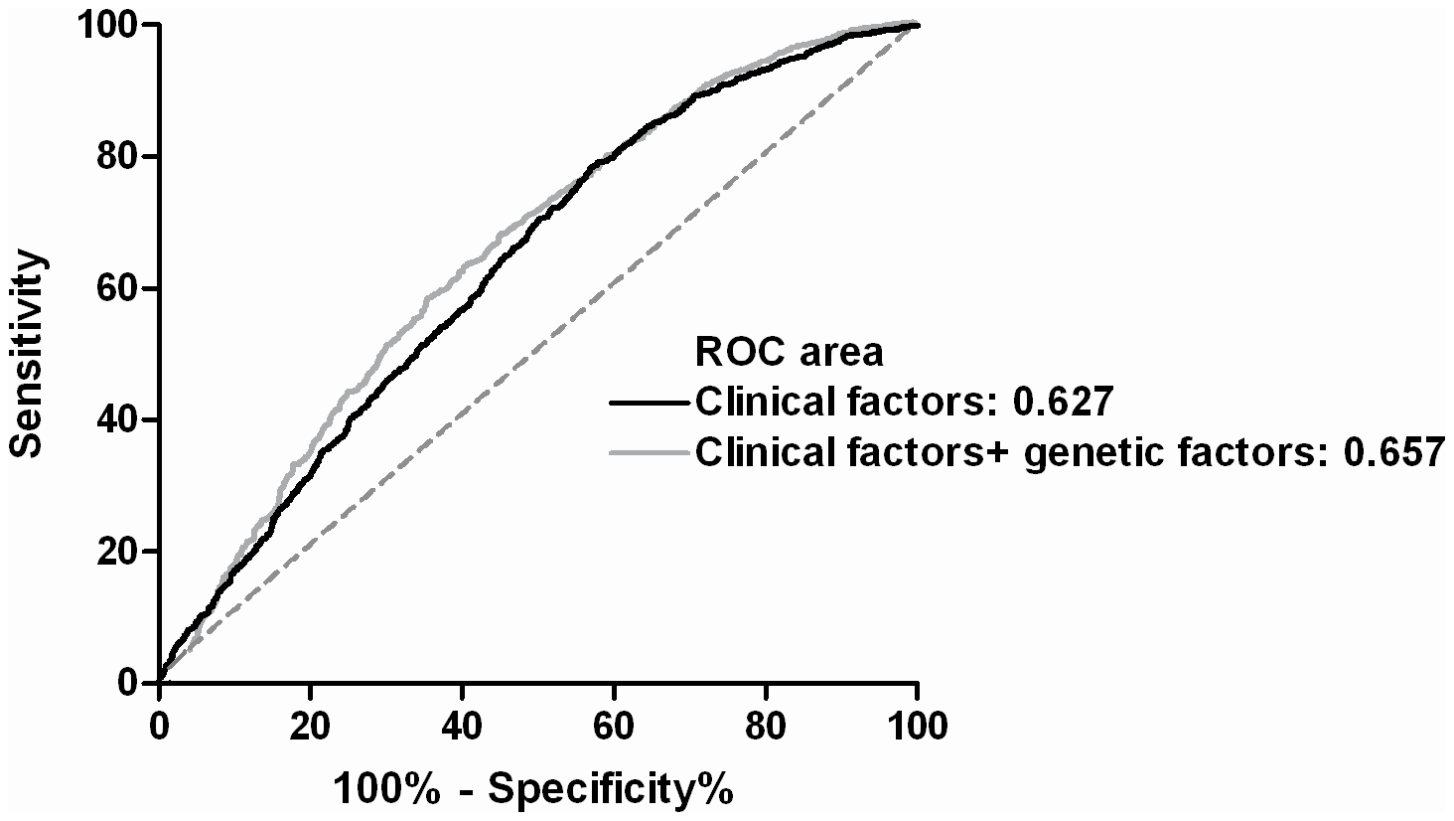
Receiver-operating characteristic curve using clinical factors (age, sex, and body mass index) (black line) or clinical factors plus genetic scores (gray line) for discrimination of type 2 diabetes.

To examine the cumulative effect of risk variant on diabetes risk, we generated weighted genotype score for each risk allele from logistic regression model. All participants were divided into four equal groups according to genotype scores (<29, 29–31, 32–34, and >34). The corresponding OR for each group compared with the lowest quartile groups are 1.26 (95% CI, 1.028–1.54, *P* = 0.02), 1.31 (95% CI, 1.072–1.61, *P* = 0.006), and 2.22 (95% CI, 1.81–2.73, *P*<0.001), respectively (Figure 2). We then tested whether the incorporation of genotype score improve the prediction of T2DM risk. The C statistics of the regression model using clinical factors including age, sex, and BMI is 0.627 (95% CI, 0.606–0.650). The incorporation of genotype score increased the C statistics to 0.657 (95% CI, 0.636–0.678) (*P*<0.0001) (Figure 3).

### Gene-gene interactions and interactions between genetic variants and other known T2DM risk factors

**Figure 4:**
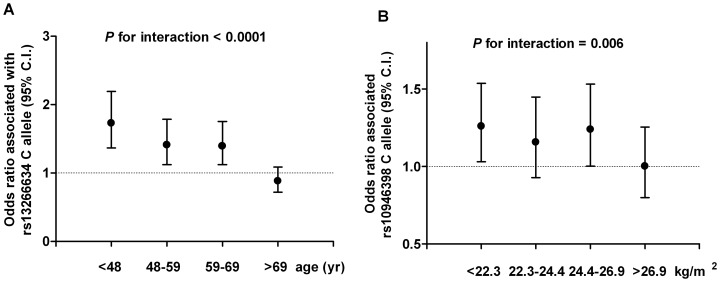
Odds ratios of type 2 diabetes associated with the risk C allele at rs13266634 in the *SLC30A8* gene according to age groups (A). Odds ratio association with type 2 diabetes associated with the C allele at rs10946398 in the *CDKAL1* gene according to body mass index groups (B).

We next explored potential gene-gene interaction and interaction with other risk factors of T2DM. No significant gene-gene interaction was found using pairwise interaction testing. Analyses of interaction between genetic variants and other known T2DM risk factors showed significant interaction between rs13266634 in *SLC30A8* gene and age on T2DM risk (*P* for interaction<0.0001, adjusted *P*<0.0001). As shown in Figure 4A, the OR associated with the risk C allele was attenuated with advanced age, ranging from 1.73 in the group of lowest age quartile (age<48) to 0.88 in the group of highest age quartile (age>69). A suggestive interaction between BMI and rs10946398 in *CDKAL1* gene (*P* for interaction = 0.006, adjusted *P* = 0.084) was also found. The increased risk associated with the C allele at rs10946398 was attenuated in subjects with larger BMI (Figure 4B). However, we did not observed significant interactions between genetic score and other T2DM risk factors including age, sex, and BMI.

### Meta-analysis

Since the statistical power of our current study was not adequate for rare alleles or alleles with small effect size, a meta-analysis pooling 20 studies in Han Chinese was conducted to further validate the association [13], [16]–[34]. As shown in Table 4 and Figure S1, 10 genetic variants in 8 genes are significantly associated with T2DM in the meta-analysis, including rs4402960 and rs1470579 in *IGF2BP2*, rs10946398 in *CDKAL1*, rs864745 in *JAZF1*, rs13266634 in *SCL30A8*, rs1111875 and rs7923837 in *HHEX*, rs7903146 in *TCF7L2*, rs1113132 in *EXT2*, and rs8050136 in *FTO* gene. The OR conferred by each risk variant did not differ significantly between Han Chinese and European populations (Figure S2A). However, substantial heterogeneity of risk allele frequencies exists between Han Chinese and European populations (Figure S2B).

**Table 4:** Meta-analysis for SNPs at twenty-four genetic loci in the European and Asian populations.

SNP	Gene	Han Chinese					Reported associations in Europeans				
		Control MAF	OR (95% CI)	*P*	N	Power (%)	Control MAF	OR (95% CI)	*P*	N	Reference
rs2641348	*ADAM30*	0.0277	1.055 (0.859–1.294)	0.61	6,635	8.6	0.107	1.10 (1.06–1.15)	**4.00E-07**	60,048	8
rs10923931	*NOTCH2*	0.029	1.027 (0.826–1.279)	0.81	5,747	5.7	0.106	1.13 (1.08–1.17)	**4.10E-08**	58,667	8
rs7578597	*THADA*	0.0046	1.300 (0.814–2.078)	0.27	6,658	22.0	0.098	1.15 (1.10–1.20)	**1.10E-09**	60,832	8
rs10490072	*BCL11A*	-	-	-	-		0.276	1.05 (1.03–1.08)	**1.00E-04**	59,682	8
rs4402960	*IGF2BP2*	0.262	1.132 (1.09–1.176)	**5.50E-09**	30,432	100	0.29	1.14 (1.11–1.18)	**8.90E-16**	32,554	4
rs1470579	*IGF2BP2*	0.276	1.109 (1.048–1.174)	**3.49E-05**	13,425	98.5	0.3	1.17 (1.11–1.23)	**1.30E-09**	13,781	4
rs17036101	*SYN2,PPARG*	-	-	-	-		0.073	1.15 (1.10–1.21)	**2.00E-07**	59,682	8
rs4607103	*ADAMTS9*	0.363	0.993(0.948–1.041)	0.78	15,389	6.1	0.239	1.09 (1.06–1.12)	**1.20E-08**	62,387	8
rs10010131	*WFS1*	0.051	1.082 (0.952–1.231)	0.23	9,370	25.2	0.4	1.11 (1.07–1.16)	**1.40E-07**	20,922	6
rs6446482	*WFS1*	0.051	1.135 (0.958–1.344)	0.14	4,955	34.1	0.41	1.11 (1.06–1.15)	**3.40E-07**	20,922	6
rs10946398	*CDKAL1*	0.448	1.206 (1.157–1.257)	**<1.0E-10**	19,705	100	0.319	1.12 (1.08–1.16)	**4.10E-11**	32,554	7
rs9472138	*VEGFA*	0.125	1.012 (0.905–1.109)	0.97	6,892	6.91	0.282	1.06 (1.04–1.09)	**4.00E-06**	63,537	8
rs864745	*JAZF1*	0.272	1.092 (1.038–1.149)	**6.98E-04**	16,996	97.0	0.499	1.10 (1.07–1.13)	**5.00E-14**	59,617	8
rs13266634	*SLC30A8*	0.421	1.160 (1.119–1.202)	**<1E-10**	26,074	100	0.306	1.12 (1.07–1.16)	**5.30E-08**	32,554	7
rs1111875	*HHEX*	0.308	1.158 (1.119–1.198)	**<1E-10**	39,986	100	0.47	1.13 (108–1.17)	**5.70E-10**	32,554	4
rs7923837	*HHEX*	0.232	1.176 (1.125–1.230)	**<1E-10**	23,051	100	0.377	1.20 (1.10–1.30)	**7.50E-06**	5,310	5
rs7903146	*TCF7L2*	0.0448	1.454 (1.258–1.680)	**1.29E-06**	10,391	100	0.26	1.37(1.31–1.43)	**1.00E-48**	32,554	4
rs7480010	*LOC387761*	0.217	0.979 (0.908–1.055)	0.58	9,007	10.0	0.301	1.17 (1.08–1.28)	**1.20E-05**	5,480	5
rs1113132	*EXT2*	0.404	1.082(1.017–1.115)	**0.013**	9,365	80.4	0.267	1.17 (1.07–1.28)	**2.90E-05**	5,404	5
rs11037909	*EXT2*	0.401	1.026 (0.965–1.092)	0.41	9,330	15.1	0.271	1.18 (1.08–1.29)	**1.30E-05**	5,379	5
rs3740878	*EXT2*	0.417	1.057(0.994–1.123)	0.078	9,345	51.3	0.272	1.17 (1.07–1.28)	**1.30E-05**	5,404	5
rs1153188	*DCD*	-	-	-	-		0.267	1.08 (1.05–1.11)	**1.80E-07**	62,301	8
rs7961581	*TSPAN8,LGR5*	0.219	1.044 (0.989–1.103)	0.12	15,487	39.6	0.269	1.09 (1.06–1.12)	**1.10E-09**	62,301	8
rs8050136	*FTO*	0.134	1.126 (1.055–1.201)	**3.24E-05**	16,690	98.3	0.398	1.17 (1.12–1.22)	**1.30E-12**	32,554	7

MAF, minor allele frequency; OR, odds ratio; 95% CI, 95% confidence interval.

## Discussion

In this study, we confirmed the association of 10 genetic risk variants identified from European GWAS in Han Chinese. The incorporation of genetic information improves the prediction of T2DM. The effect sizes conferred by risk variants are similar but the allele frequencies differ substantially between Han Chinese and European populations.

Previous GWAS in Han Chinese identified several candidate variants associated with T2DM. Tsai et al reported genetic variants in *PRPRD* and *SRR* genes associated with T2DM in a GWAS conducted in Han Chinese in Taiwan [38]. Another GWAS in Han Chinese by Shu et al found that genetic variants near *CDC123*/*CAMD1A*, *SPRY2*, and *C2CD4B* genes are associated with T2DM[28]. However, the loci discovered from both GWAS did not overlap with each other. Therefore, instead of testing these variants, we attempted to validate the association of established risk loci in Europeans in our population. Given the heterogeneous genetic structure of different ethnic populations, it is necessary to validate the relative contribution of T2DM variants identified from Caucasian GWAS in Han Chinese. Here, we confirmed the association of genetic variants in the *IGF2BP2*, *CDKAL1*, *JAZF1*, *SCL30A8*, *HHEX*, *TCF7L2*, *EXT2*, and *FTO* gene with T2DM in Han Chinese population. Interestingly, the effect sizes conferred by these variants were similar between Han Chinese and European populations despite marked differences in allele frequencies, suggesting that the biological actions of these variants are the same across different ethnic groups. The effect size of several uncommon or rare variants, including rs10490072 in the *BCL11A* gene, rs7578597 in the *THADA* gene, and rs7903146 in the *TCF7L2* gene, are relatively large (OR: 1.34, 1.30, and 1.45, respectively). Therefore an aggregate of all SNPs were used for our prediction model of T2DM instead of using only common variants.

We found that the addition of genetic information to clinical predictors slightly improved the prediction for T2DM in Han Chinese. The 3% increment in C-statistics is consistent with those observed in Europeans [10], [11]. Similarly, Xu et al reported that a 1.6% increase in C-statistics for T2DM prediction in another Chinese population using a set of 19 risk variants. Collectively, these studies confirmed a small improvement in the prediction of T2DM by incorporating genetic information. It should be noted that such increment in C-statistics, albeit statistically significant, may not be of clinical significance. However, we also found that the OR of T2DM in subjects with the highest genetic score quartile was 2.22 as compared with those with the lowest genetic score quartile. This estimate is in concordance with the 2.60-fold increased risk associated with higher genetic scores in a European population as reported by Meigs et al. [11].

An interesting interaction was found between age and an exonic variant in the *SLC30A8* gene. The S*LC30A8* gene encodes a zinc transporter specifically expressed in pancreatic beta-cells. We found that the effect conferred by the risk allele was diminished with aging. The underlying mechanism is currently unknown. Zinc deficiency has been shown to develop with advanced age when the ability to transport zinc is disrupted [39]. Therefore, the reduced zinc transporter capability associated with aging may mask the genetic effect of S*LC30A8* mutation. However, further replication is needed to verify this observation.

Our study has both strengths and limitations which need to be addressed. First, this study provides the largest and the most updated meta-analysis for T2DM genetic association in Han Chinese population. However, this study are still insufficiently powered to validate the association of variants in *ADAM30*, *NOTCH2*, *THADA*, *ADAMTS9, WFS1, VEGFA, LOC387761*, and *TSPAN8/LGR5* genes with T2DM, probably owing to their low allele frequencies and small effects in Han Chinese. Second, variants identified by recent GWAS in East Asians were not genotyped [40]–[43]. With the rapidly expanding knowledge for T2DM genetics, further incorporation of new genetic variants is warranted for in order to enhance prediction. Third, this study could not provide accurate estimation of disease incidence because of the case-control design. Therefore, the net improvement in re-classification could not be estimated.

In summary, this study affirmed the association of 10 genetic loci with T2DM in Han Chinese. Carriers with higher genetic risk scores have a 2.2-fold increase in T2DM risk and the addition of genetic information to clinical factors lead to a ∼3% increment in the discriminative power for prediction of T2DM. These data, together with previous studies, support the usefulness of genetic testing for T2DM prediction.

## Supporting Information

Figure S1 **Forest plots for meta-analyses showing odds ratios of type 2 diabetes conferred by risk variants identified from European genome-wide association studies in Han Chinese.**
(DOC)

Figure S2 **Comparison of odds ratio associated with risk alleles (A) or minor allele frequencies (B) between Chinese and European populations.**
(TIF)

Checklist S1 PRISMA (Preferred Reporting Items for Systemic Review and Meta-analysis) check-list and flow diagram for meta-analysis.
(DOC)
