# Supplementary material for: Validation of Type 2 Diabetes Risk Variants Identified by Genome-Wide Association Studies in Han Chinese Population: A Replication Study and Meta-Analysis
Source: PLoS One. 2014 Apr 15;9(4):e95045. doi: 10.1371/journal.pone.0095045 (PMC3988150; doi:10.1371/journal.pone.0095045)

**Figure S1.** Forest plots for meta-analyses showing odds ratios of type 2 diabetes conferred by risk variants identified from European genome-wide association studies in Han Chinese

rs2641348 *ADAM30*


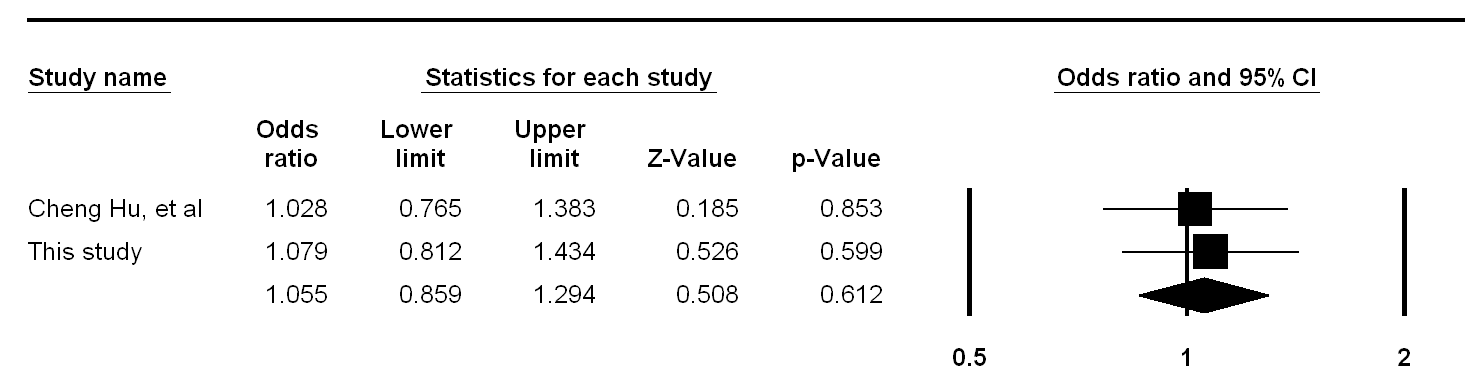


rs10923931 *NOTCH2*


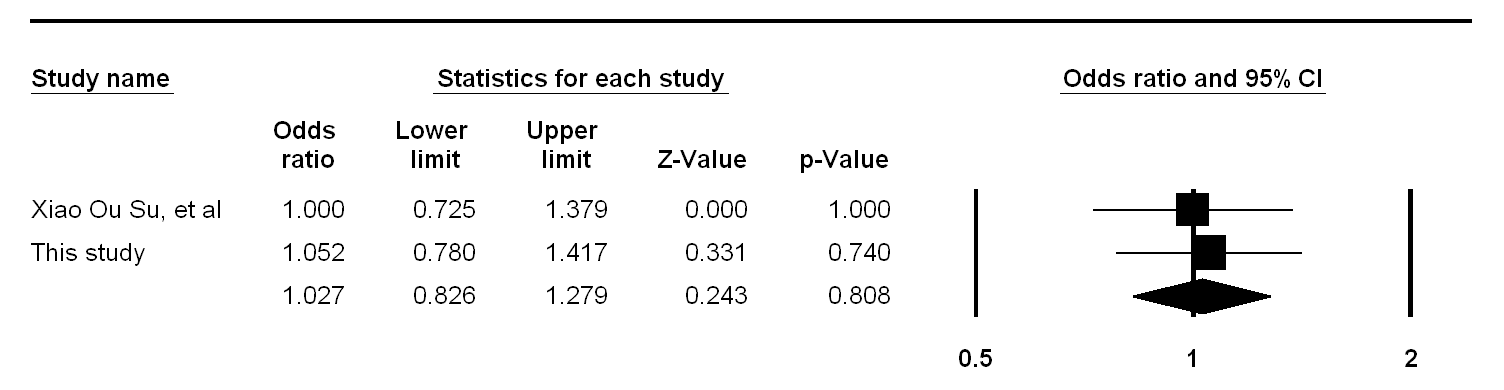


rs7578597 *THADA*


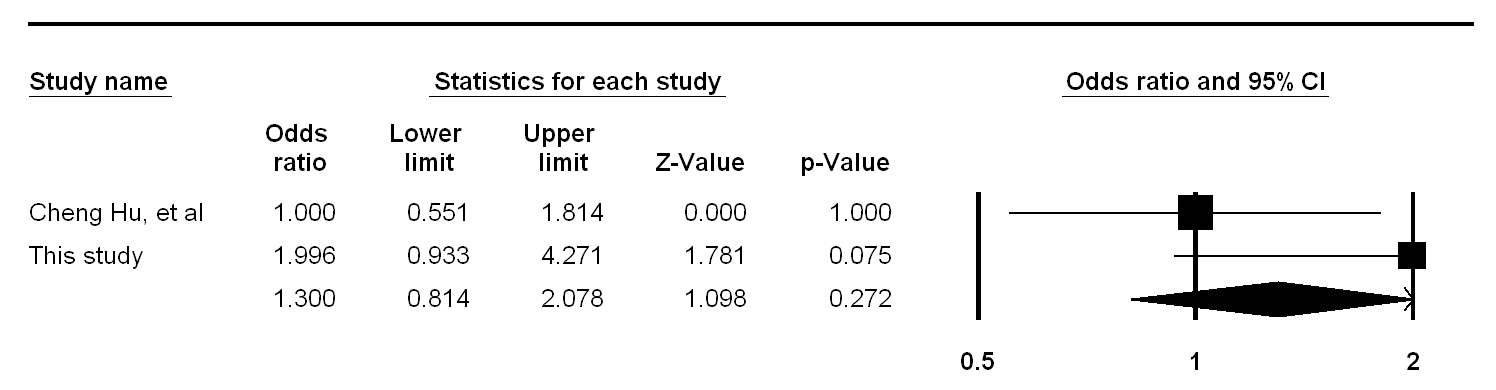


rs4402960 *IGF2BP2*


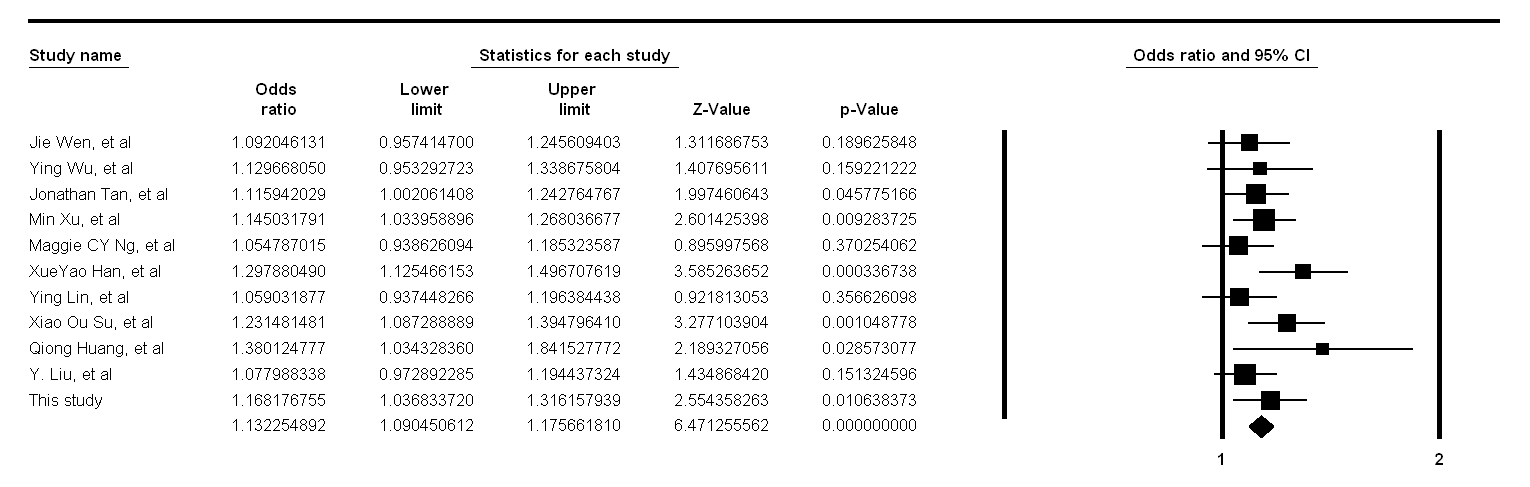


rs1470579 *IGF2BP2*


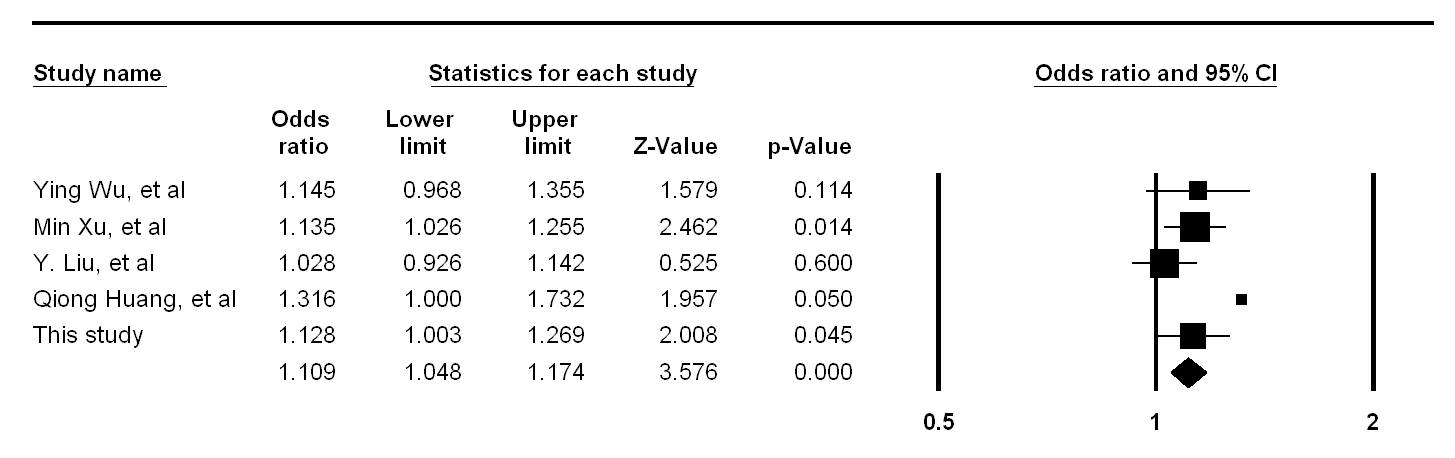


rs4607103 *ADAMTS9*


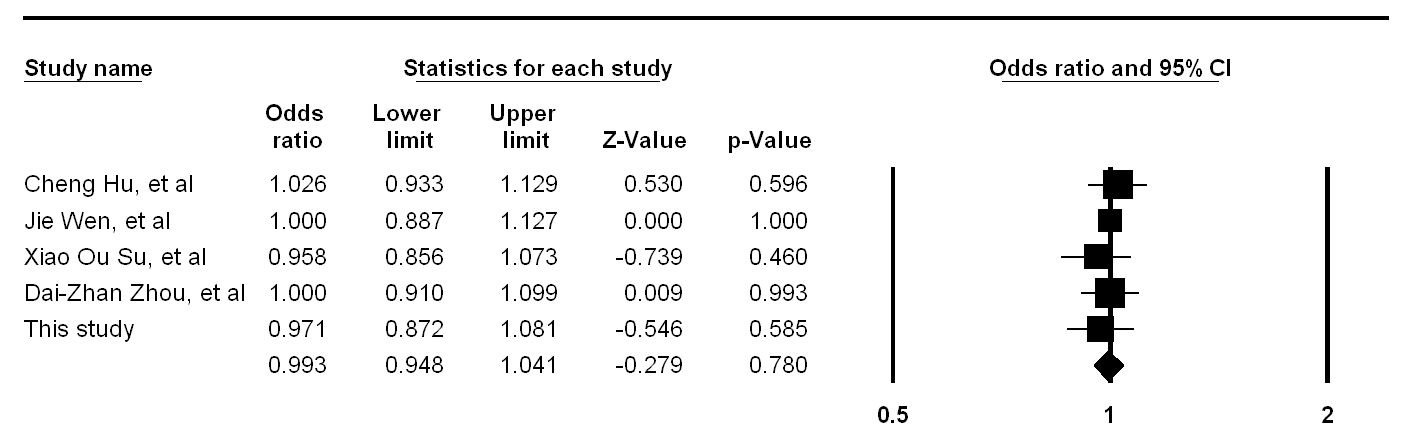


rs10010131 *WFS1*


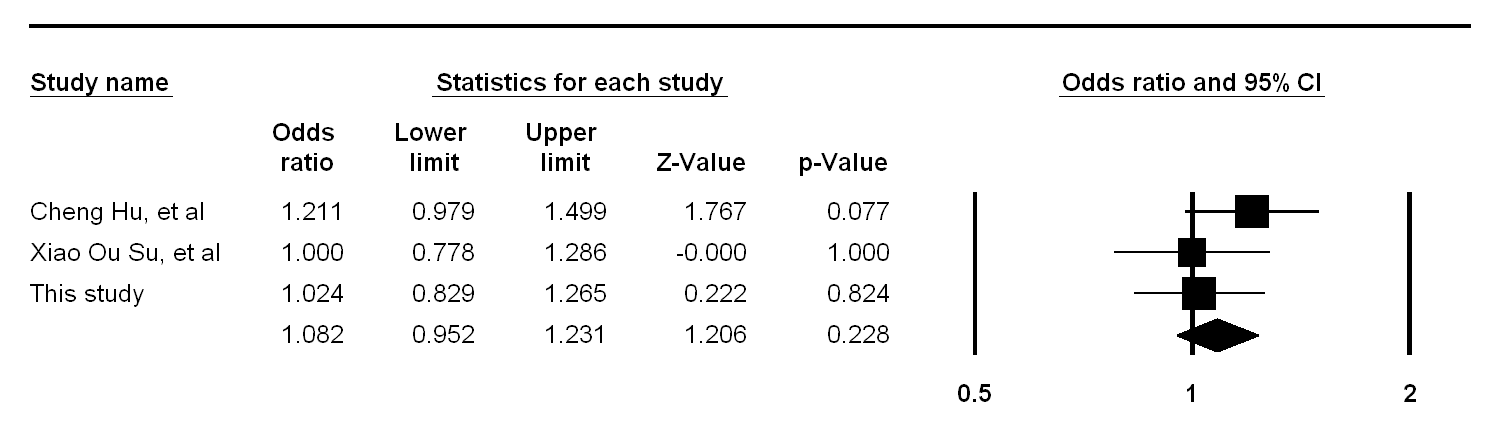


rs6446482 *WFS1*


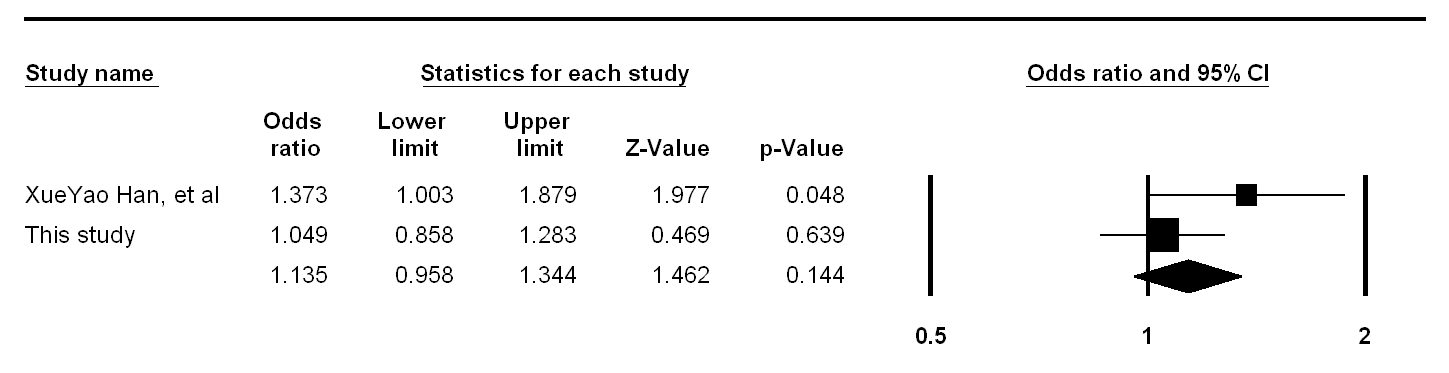


rs10946398 *CDKAL1*


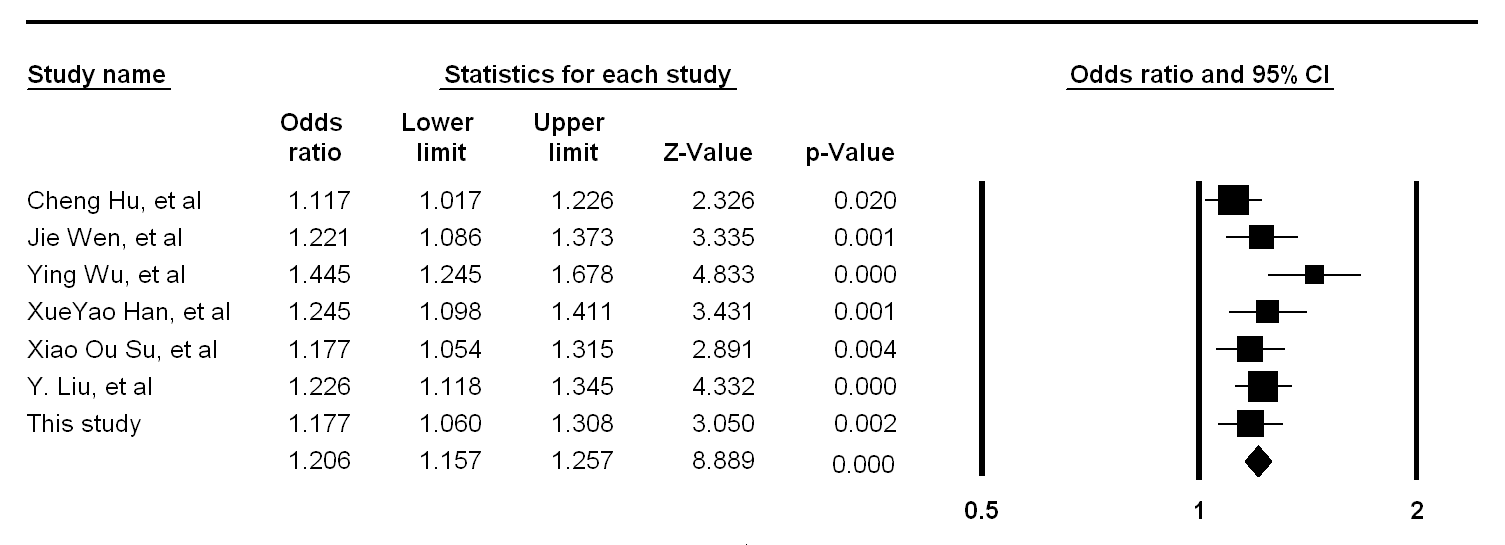


rs9472138 *VEGFA*


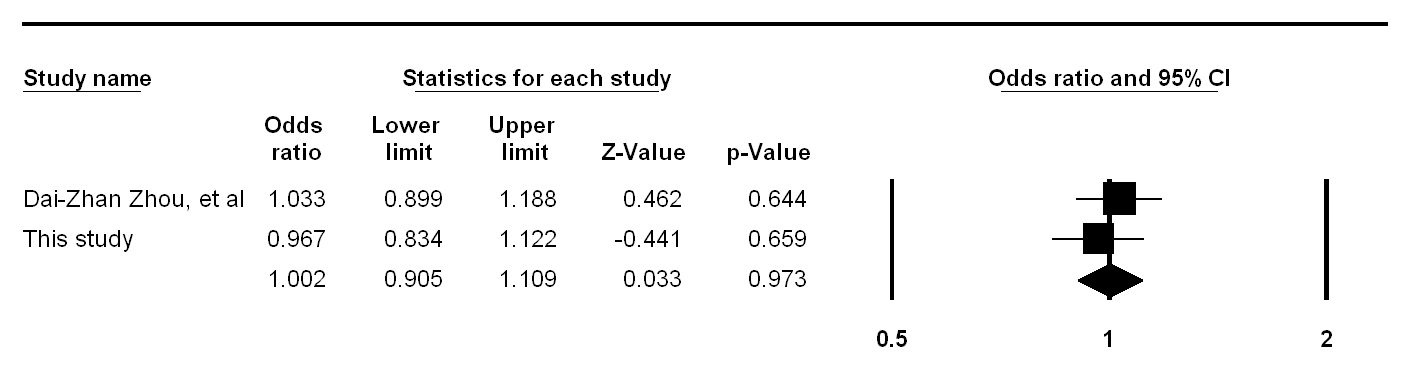


rs864745 *JAZF1*


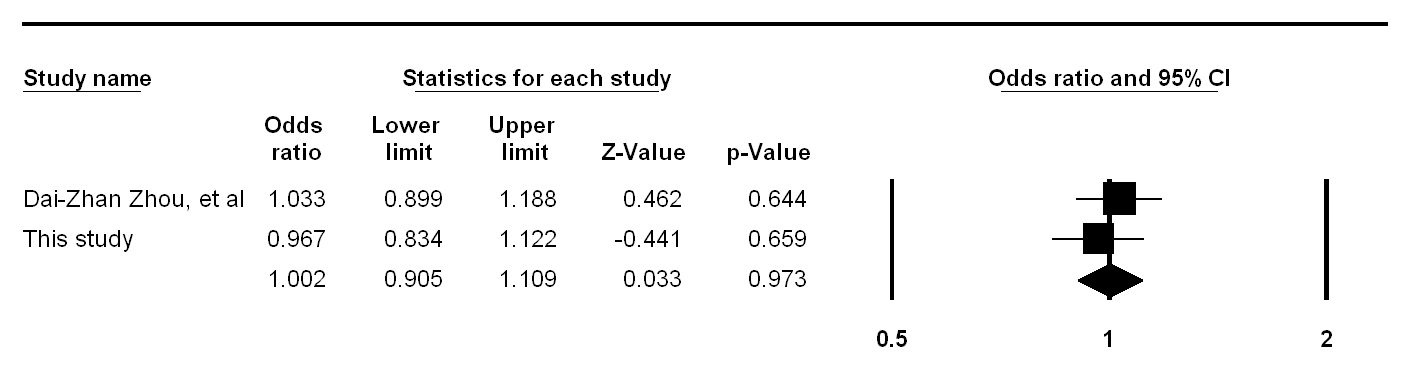


rs13266634 *SLC30A8*


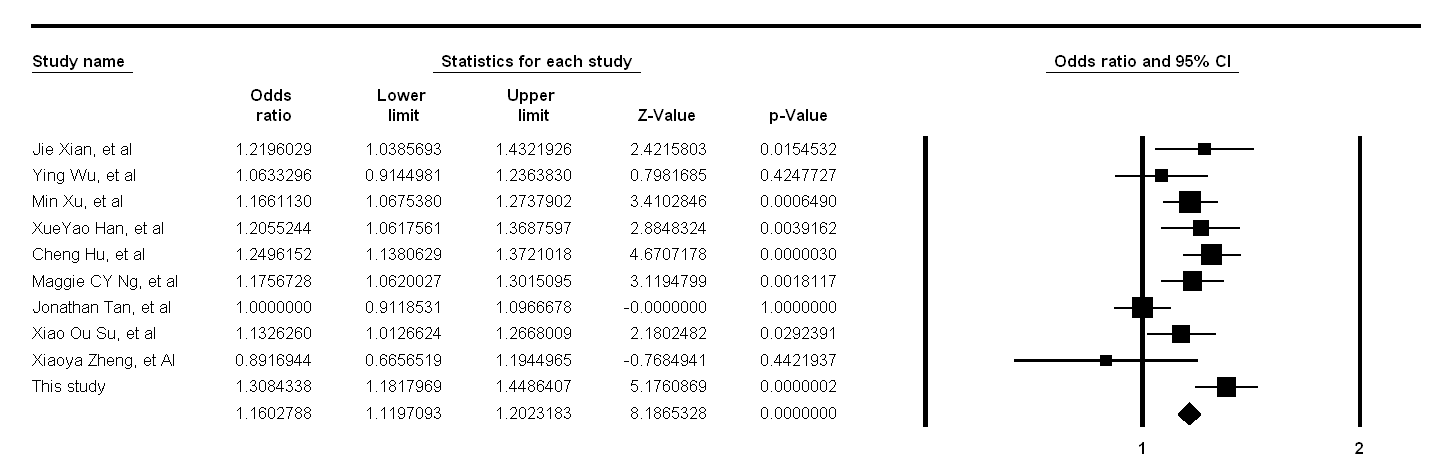


rs1111875 *HHEX*


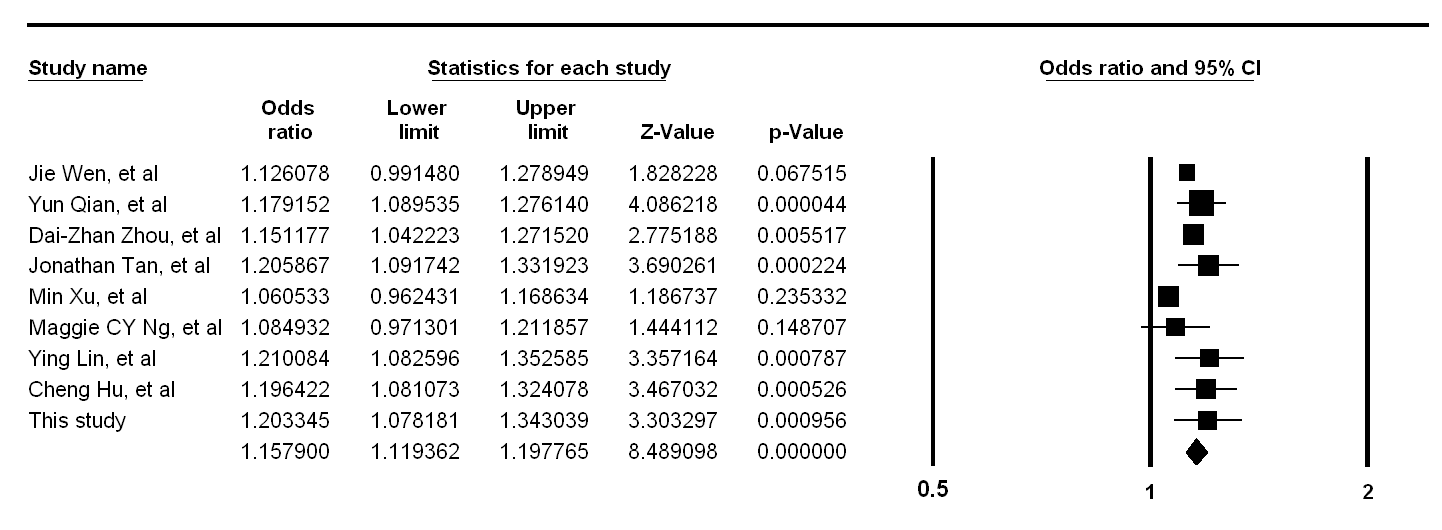


rs7923837 *HHEX*


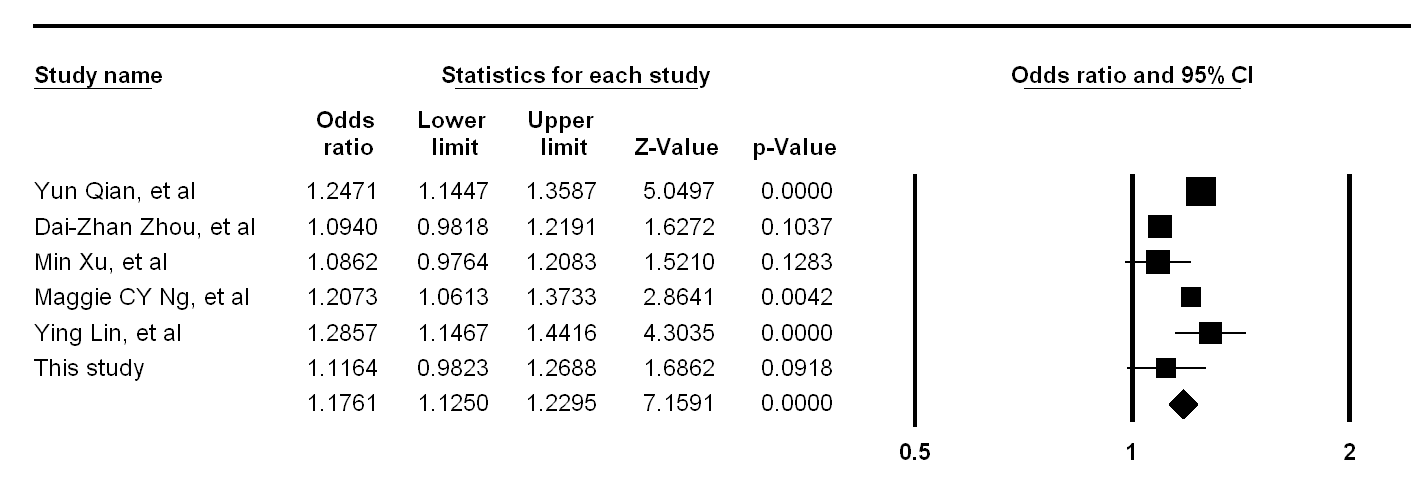


rs7903146 *TCF7L2*


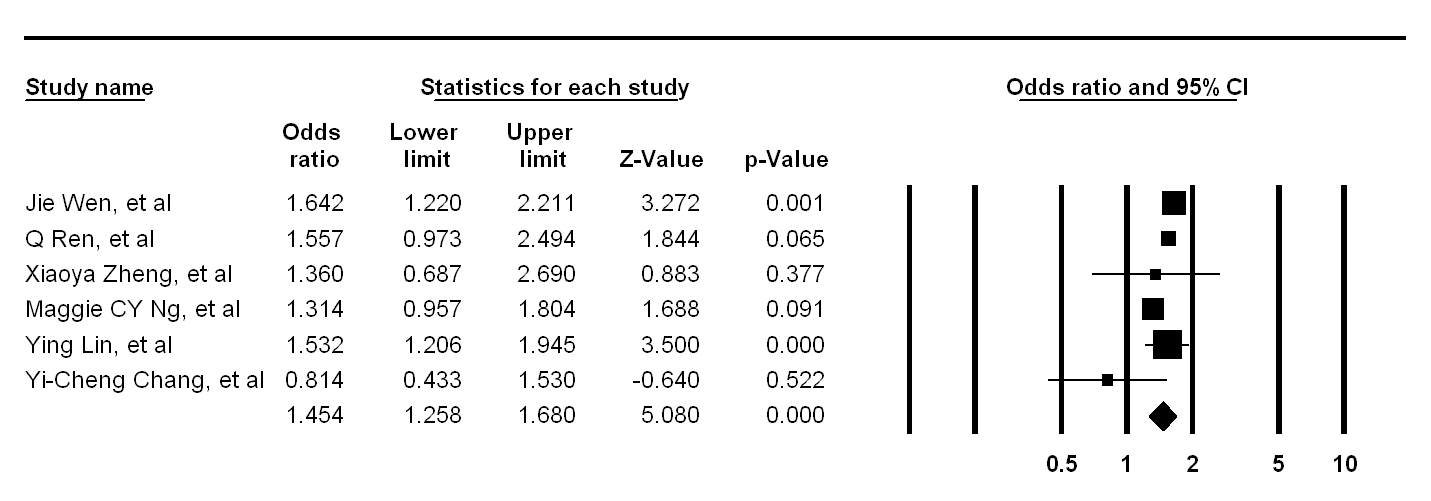


rs7480010 *LOC387761*


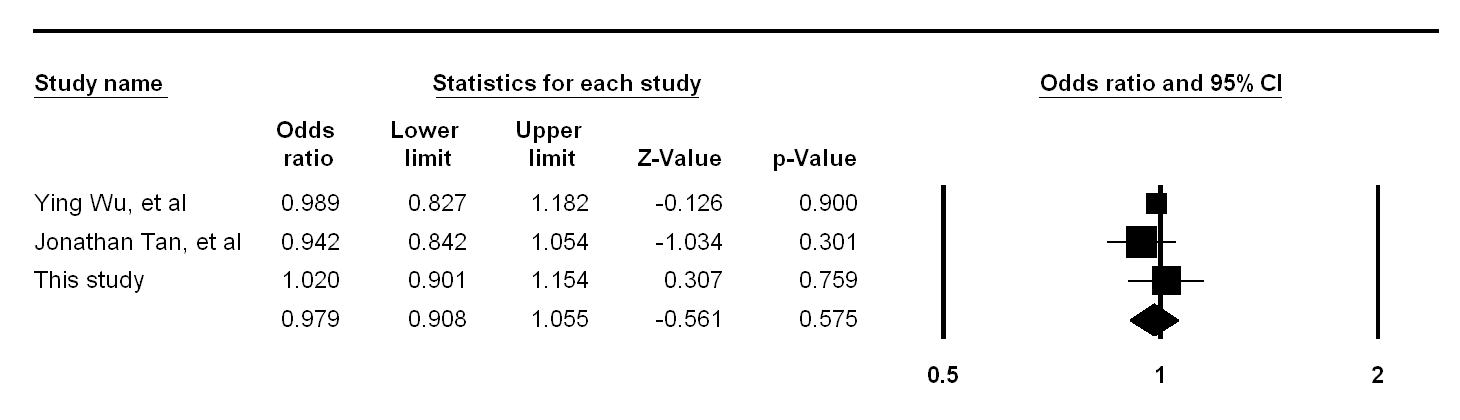


rs1113132 *EXT2*


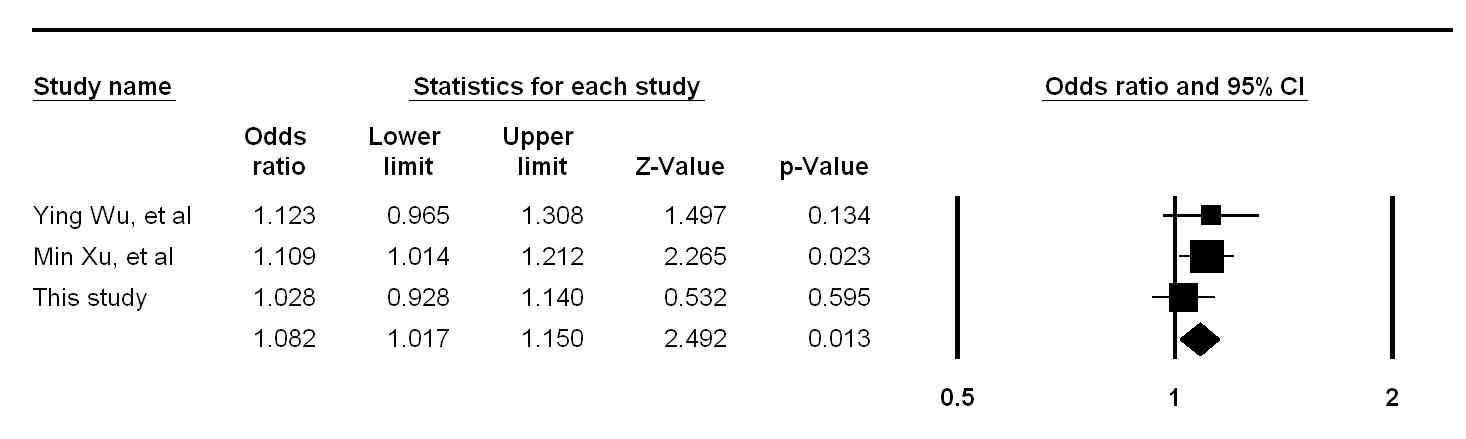


rs11037909 *EXT2*


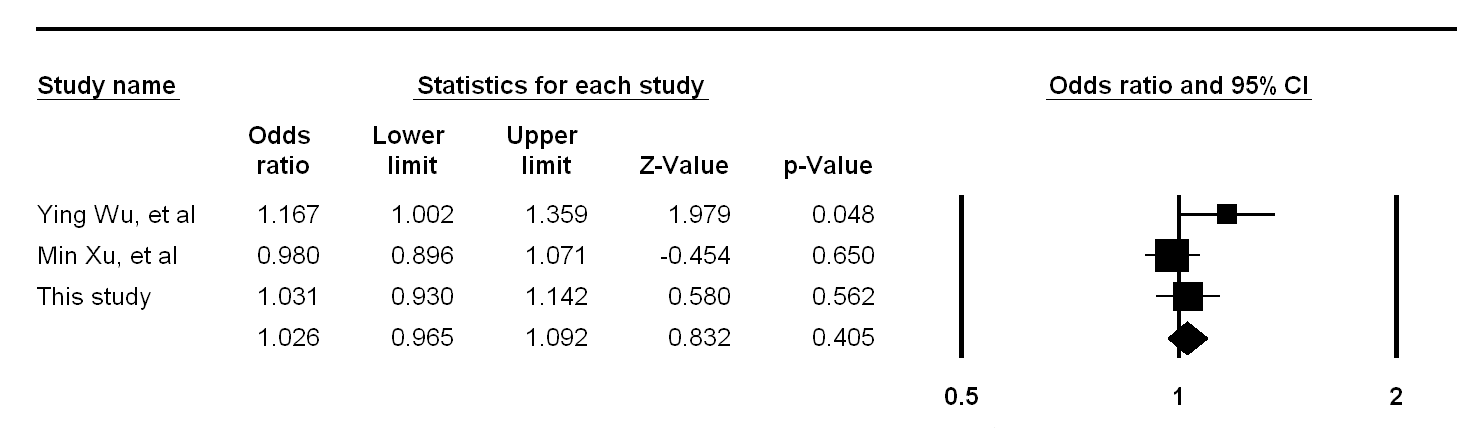


rs3740878 *EXT2*


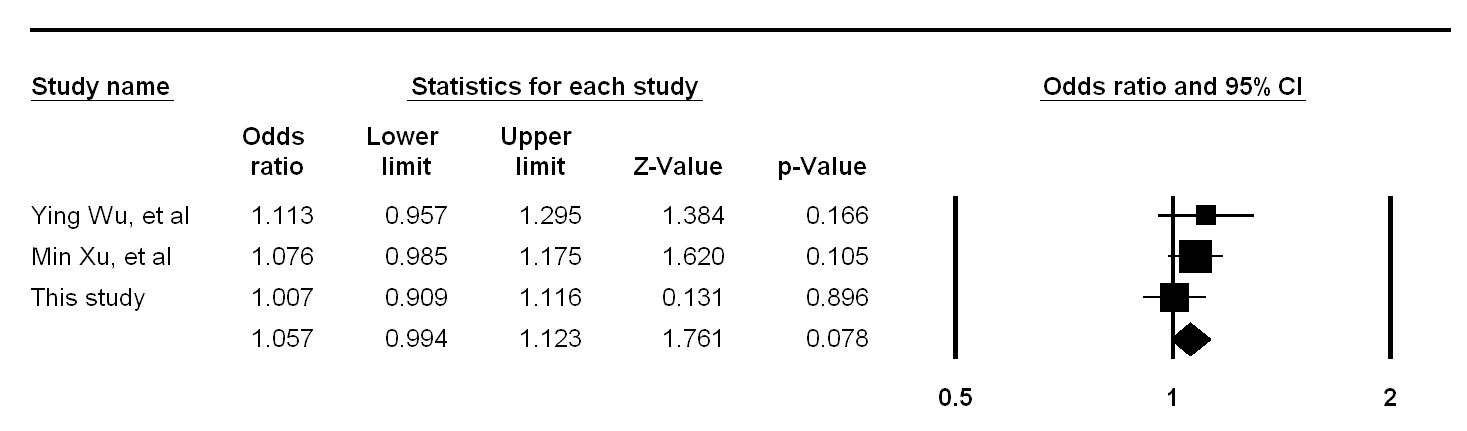


rs7961581 *TSPAN8,LGR5*


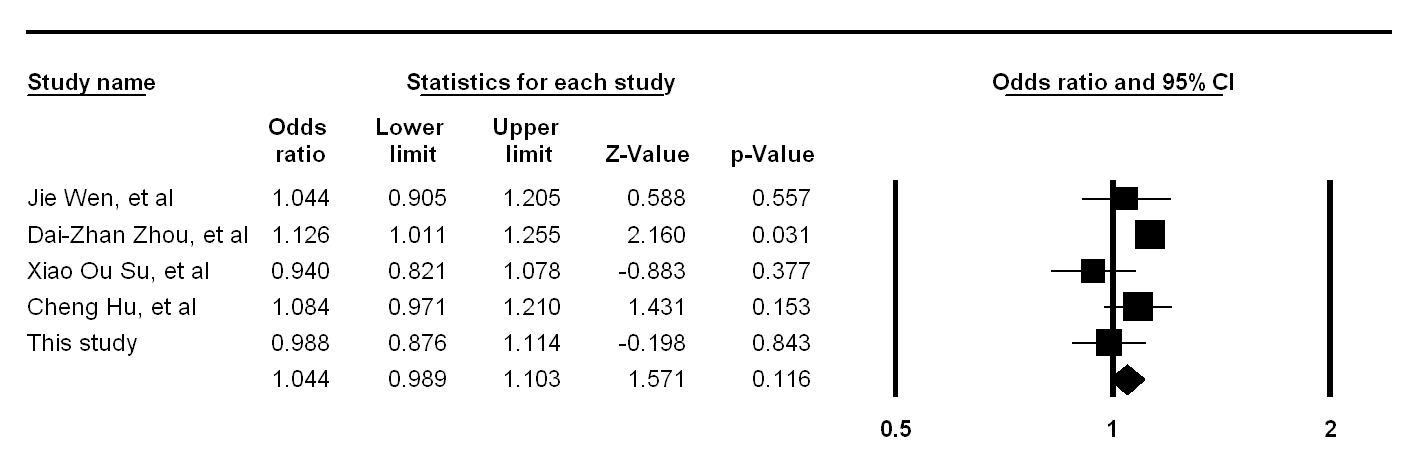


rs8050136 *FTO*


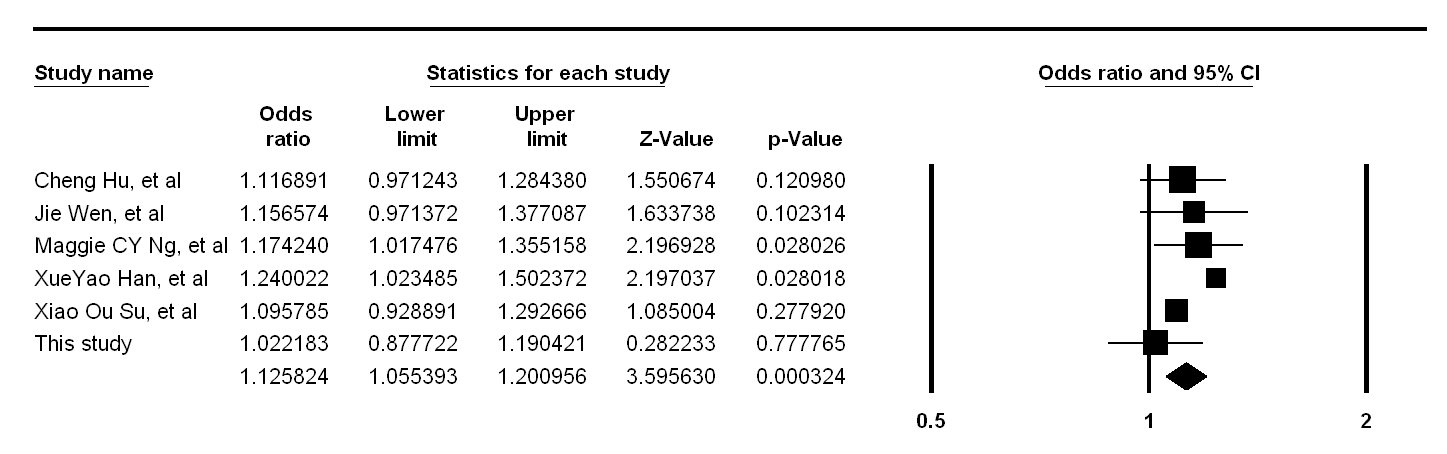

Supplement: Figure S1 — Forest plots for meta-analyses showing odds ratios of type 2 diabetes conferred by risk variants identified from European genome-wide association studies in Han Chinese. (DOC) [file pone.0095045.s001.doc]
