# Supplementary figures and images for: Validation of Type 2 Diabetes Risk Variants Identified by Genome-Wide Association Studies in Han Chinese Population: A Replication Study and Meta-Analysis
Source: PLoS One. 2014 Apr 15;9(4):e95045. doi: 10.1371/journal.pone.0095045 (PMC3988150; doi:10.1371/journal.pone.0095045)

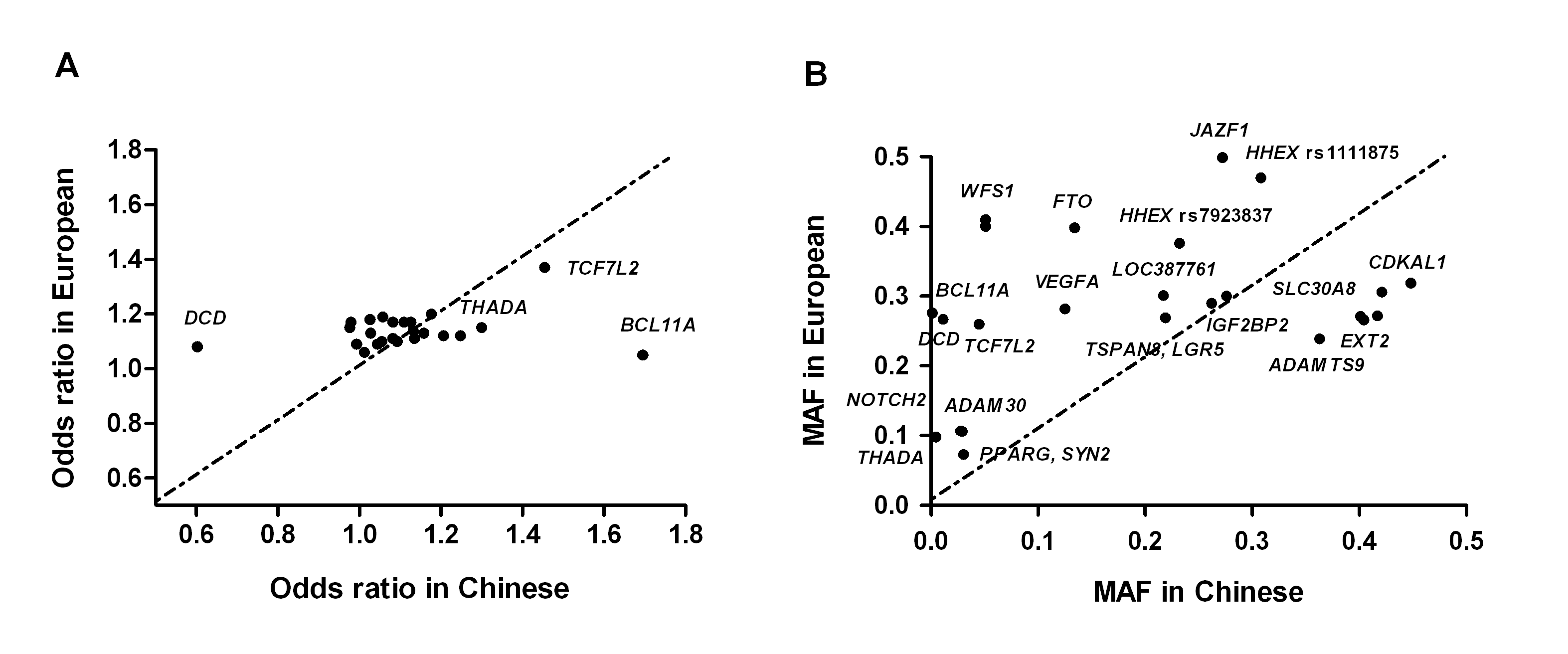

Supplement: Figure S2 — Comparison of odds ratio associated with risk alleles (A) or minor allele frequencies (B) between Chinese and European populations. (TIF) [file pone.0095045.s002.tif]
